# Drug characteristics derived from kinetic modeling: combined ^11^C-UCB-J human PET imaging with levetiracetam and brivaracetam occupancy of SV2A

**DOI:** 10.1186/s13550-022-00944-5

**Published:** 2022-11-08

**Authors:** Mika Naganawa, Jean-Dominique Gallezot, Sjoerd J. Finnema, Ralph Paul Maguire, Joël Mercier, Nabeel B. Nabulsi, Sophie Kervyn, Shannan Henry, Jean-Marie Nicolas, Yiyun Huang, Ming-Kai Chen, Jonas Hannestad, Henrik Klitgaard, Armel Stockis, Richard E. Carson

**Affiliations:** 1 Yale University School of Medicine, 801 Howard Ave, PO Box 208048, New Haven, CT USA; 2 UCB Pharma, Braine-l’Alleud, Belgium; 3 Tranquis Therapeutics, Inc, Redwood City, CA USA

**Keywords:** Positron emission tomography, Kinetic modeling, SV2A, Occupancy, Displacement

## Abstract

**Background:**

Antiepileptic drugs, levetiracetam (LEV) and brivaracetam (BRV), bind to synaptic vesicle glycoprotein 2A (SV2A). In their anti-seizure activity, speed of brain entry may be an important factor. BRV showed faster entry into the human and non-human primate brain, based on more rapid displacement of SV2A tracer ^11^C-UCB-J. To extract additional information from previous human studies, we developed a nonlinear model that accounted for drug entry into the brain and binding to SV2A using brain ^11^C-UCB-J positron emission tomography (PET) data and the time-varying plasma drug concentration, to assess the kinetic parameter *K*_1_ (brain entry rate) of the drugs.

**Method:**

Displacement (LEV or BRV p.i. 60 min post-tracer injection) and post-dose scans were conducted in five healthy subjects. Blood samples were collected for measurement of drug concentration and the tracer arterial input function. Fitting of nonlinear differential equations was applied simultaneously to time-activity curves (TACs) from displacement and post-dose scans to estimate 5 parameters: *K*_1_ (drug), *K*_1_(^11^C-UCB-J, displacement), *K*_1_(^11^C-UCB-J, post-dose), free fraction of ^11^C-UCB-J in brain (*f*_ND_(^11^C-UCB-J)), and distribution volume of ^11^C-UCB-J (*V*_T_(UCB-J)). Other parameters (*K*_D_(drug), *K*_D_(^11^C-UCB-J), *f*_P_(drug), *f*_P_(^11^C-UCB-J, displacement), *f*_P_(^11^C-UCB-J, post-dose), *f*_ND_(drug), *k*_off_(drug), *k*_off_(^11^C-UCB-J)) were fixed to literature or measured values.

**Results:**

The proposed model described well the TACs in all subjects; however, estimates of drug *K*_1_ were unstable in comparison with ^11^C-UCB-J *K*_1_ estimation. To provide a conservative estimate of the relative speed of brain entry for BRV vs. LEV, we determined a lower bound on the ratio BRV *K*_1_/LEV *K*_1_, by finding the lowest BRV *K*_1_ or highest LEV *K*_1_ that were statistically consistent with the data. Specifically, we used the *F* test to compare the residual sum of squares with fixed BRV *K*_1_ to that with floating BRV *K*_1_ to obtain the lowest possible BRV *K*_1_; the same analysis was performed to find the highest LEV *K*_1_. The lower bound of the ratio BRV *K*_1_/LEV *K*_1_ was ~ 7.

**Conclusions:**

Under appropriate conditions, this advanced nonlinear model can directly estimate entry rates of drugs into tissue by analysis of PET TACs. Using a conservative statistical cutoff, BRV enters the brain at least sevenfold faster than LEV.

**Supplementary Information:**

The online version contains supplementary material available at 10.1186/s13550-022-00944-5.

## Background

The antiepileptic drugs (AEDs) levetiracetam (LEV) and brivaracetam (BRV) bind to the synaptic vesicle glycoprotein 2A (SV2A). BRV has an indication for focal onset seizure in several regions including the EU and US and has a chemical structure related to that of approved AED, LEV, but with 15–30-fold higher affinity for SV2A than LEV based on in vitro studies [1–3]. The kinetic properties of BRV and LEV in brain tissue have been assessed in mice, non-human primates, and humans. These studies consistently suggest that the rate of brain penetration and the onset of action are faster for BRV than LEV [1, 4]. These kinetic properties, especially the speed of entry into the brain, may play an important role in the treatment of acute epileptic seizures.

In previous studies in non-human primates and humans, we performed dynamic positron emission tomography (PET) imaging with ^11^C-UCB-J, a SV2A radiotracer and intravenous infusion of either BRV or LEV to displace ^11^C-UCB-J binding. BRV entered the brain faster than LEV, as determined by the more rapid displacement of ^11^C-UCB-J binding. The half-time of ^11^C-UCB-J signal change was computed by applying a single exponential model to the displacement measurements, and indicated a faster displacement by BRV than LEV in non-human primates and humans [1, 4]. Since overall reduction in the observed tracer binding signal requires drug entry into the brain, binding to SV2A, and tracer exit, a corrected tracer displacement half-time (i.e., drug entry to brain half-time) was estimated by subtracting the maximum tracer clearance half-time from the tracer displacement half-time.

In the current study, we developed a nonlinear mathematical model that describes the relationship between human brain ^11^C-UCB-J PET data and the time-varying drug concentration in the plasma to extract maximum information from these displacement studies. In a similar manner to the tracer, this model explicitly accounts for the uptake and binding of the AED. Because this model has a large number of parameters, many were fixed based on the literature or direct measurement to produce a model with fewer parameters to estimate. The kinetic parameters of the AEDs and ^11^C-UCB-J, especially *K*_1_ (i.e., speed of entry into brain tissue), were estimated and compared to directly quantify the relative brain uptake rates of BRV and LEV.

## Methods

### Study design and human subjects

Dynamic ^11^C-UCB-J PET scans with serial arterial sampling data [4] were reanalyzed. The details of ^11^C-UCB-J PET and arterial plasma data acquisitions were the same as described previously [5]. Five healthy subjects participated in a displacement and post-dose measurement. Four subjects each underwent a total of four PET scans, 2 each with BRV and LEV. One subject had only two PET scans (BRV displacement and post-dose). ^11^C-UCB-J was administered in a bolus plus infusion protocol [6] (*K*_bol_ = 150 min). In the first PET scan of the day, either BRV (50 mg, *n* = 1; 100 mg, *n* = 2; 200 mg, *n* = 2) or LEV (1500 mg, *n* = 4) was intravenously administered over 5 min starting at 60 min (80 min for BRV displacement for one subject) after tracer injection. Doses considered to be equipotent, intermediate doses, within the therapeutic range were chosen: 100 mg BRV and 1500 mg LEV, which were thought to be roughly equipotent [7, 8]. Then, the second post-dose PET scan was performed 3.5 ± 0.8 h after the end of the displacement scan and 4.5 ± 0.8 h after drug administration.

As part of the subject evaluation, magnetic resonance (MR) images were acquired on all subjects to eliminate those with structural brain abnormalities and for PET image registration. MR imaging was performed on a 3T whole-body scanner (TrioTrim, Siemens Medical Solutions) with a circularly polarized head coil.

### PET imaging experiments

^11^C-UCB-J was synthesized as previously described [9]. PET scans were conducted on the High-Resolution Research Tomograph (HRRT) (Siemens Medical Solutions, Knoxville, TN, USA), which acquires 207 slices (1.2-mm slice separation) with a reconstructed image resolution of ~ 3 mm at full width at half maximum. Prior to tracer administration, a 6-min transmission scan was conducted for attenuation correction. Each scan was acquired in list mode for 120 min. Dynamic scans were reconstructed in 33 frames (6 × 0.5 min, 3 × 1 min, 2 × 2 min, 22 × 5 min) with corrections for attenuation, normalization, scatter, randoms, and deadtime using the MOLAR algorithm [10]. Event-by-event motion correction [11] was included in the reconstruction based on measurements with the Polaris Vicra sensor (NDI Systems, Waterloo, Canada) with reflectors mounted on a swim cap worn by the subject. The dynamic PET images were co-registered to the early summed PET images (0–10 min post-injection) using a 6-parameter mutual information algorithm [12] (FLIRT, FSL) to eliminate any residual motion.

### PET image analysis

The putamen, frontal cortex, and cerebellum regions of interest (ROIs) were taken from the Automated Anatomical Labeling (AAL) for SPM2 [13] in Montreal Neurological Institute (MNI) space [14]. An average PET image (0–10 min) was co-registered to the subject’s T1-weighted 3T MR image (6-parameter rigid registration), which was subsequently co-registered to the AAL template in MNI space using a nonlinear transformation (Bioimage suite) [15]. Using the combined transformations from template to PET space, regional time-activity curves (TACs) were generated.

### Input function measurement

For each subject, the radial artery was catheterized for blood sampling. Arterial blood samples were collected manually every 10 s for the first 90 s and at 1.75, 2, 2.25, 2.75, 3, 4, 5, 6, 8, 10, 15, 20, 25, 30, 45, 60, 75, 90, 105, and 120 min after ^11^C-UCB-J injection for measurement of the arterial input function.

Plasma was obtained by centrifugation at 4 °C (2930 g for 5 min). Whole blood and plasma were counted in cross-calibrated gamma counters (1480 & 2480 WIZARD, PerkinElmer, Waltham, MA, USA). Analysis of the metabolite profile in the arterial plasma samples was performed using an automatic column-switching high-performance liquid chromatography system. Arterial blood samples taken immediately prior to tracer injection were used for analysis of plasma free fraction (*f*_P_) using an ultrafiltration method. The details of blood processing method were described in our previous publication [5].

Additional arterial blood samples were collected for measurement of BRV or LEV plasma concentrations at 1, 3, 5, 6, 8, 10, 15, 20, 30, 45, 60 min after the start of drug infusion in the displacement PET scans, and immediately before, in the middle, and at the end of the post-drug PET scans. These AED blood concentrations were interpolated linearly and used as the drug arterial input function.

### Quantitative analysis

In addition to conventional tracer kinetic modeling using linear compartment models, an advanced nonlinear model was also applied accounting for drug entry into the brain and SV2A binding using the drug input function. Based on the compartment models proposed in Delforge et al. [16] and Endres et al. [17], the model for tracer and drug is described as follows:

1 $$\frac{{{\text{d}}C_{{{\text{ND}}}} \left( t \right)}}{{{\text{d}}t}} = K_{1} C_{{\text{P}}} \left( t \right) - k_{2} C_{{{\text{ND}}}} \left( t \right) - f_{{{\text{ND}}}} k_{{{\text{on}}}} \left( {B_{{{\text{max}}}} - \frac{{C_{{\text{S}}} \left( t \right)}}{M} - D_{{\text{S}}} \left( t \right)} \right)C_{{{\text{ND}}}} \left( t \right) + k_{{{\text{off}}}} C_{{\text{S}}} \left( t \right),$$

2 $$\frac{{{\text{d}}C_{{\text{S}}} \left( t \right)}}{{{\text{d}}t}} = f_{{{\text{ND}}}} k_{{{\text{on}}}} \left( {B_{{{\text{max}}}} - \frac{{C_{{\text{S}}} \left( t \right)}}{M} - D_{{\text{S}}} \left( t \right)} \right)C_{{{\text{ND}}}} \left( t \right) - k_{{{\text{off}}}} C_{{\text{S}}} \left( t \right),$$

3 $$\frac{{{\text{d}}D_{{{\text{ND}}}} \left( t \right)}}{{{\text{d}}t}} = K_{1}^{{\text{D}}} D_{{\text{P}}} \left( t \right) - k_{2}^{{\text{D}}} D_{{{\text{ND}}}} \left( t \right) - f_{{{\text{ND}}}}^{{\text{D}}} k_{{{\text{on}}}}^{{\text{D}}} \left( {B_{{{\text{max}}}} - \frac{{C_{{\text{S}}} \left( t \right)}}{M} - D_{{\text{S}}} \left( t \right)} \right)D_{{{\text{ND}}}} \left( t \right) + k_{{{\text{off}}}}^{{\text{D}}} D_{{\text{S}}} \left( t \right),$$

4 $$\frac{{{\text{d}}D_{{\text{S}}} \left( t \right)}}{{{\text{d}}t}} = f_{{{\text{ND}}}}^{{\text{D}}} k_{{{\text{on}}}}^{{\text{D}}} \left( {B_{{{\text{max}}}} - \frac{{C_{{\text{S}}} \left( t \right)}}{M} - D_{{\text{S}}} \left( t \right)} \right)D_{{{\text{ND}}}} \left( t \right) - k_{{{\text{off}}}}^{{\text{D}}} D_{{\text{S}}} \left( t \right).$$

**Fig. 1:**
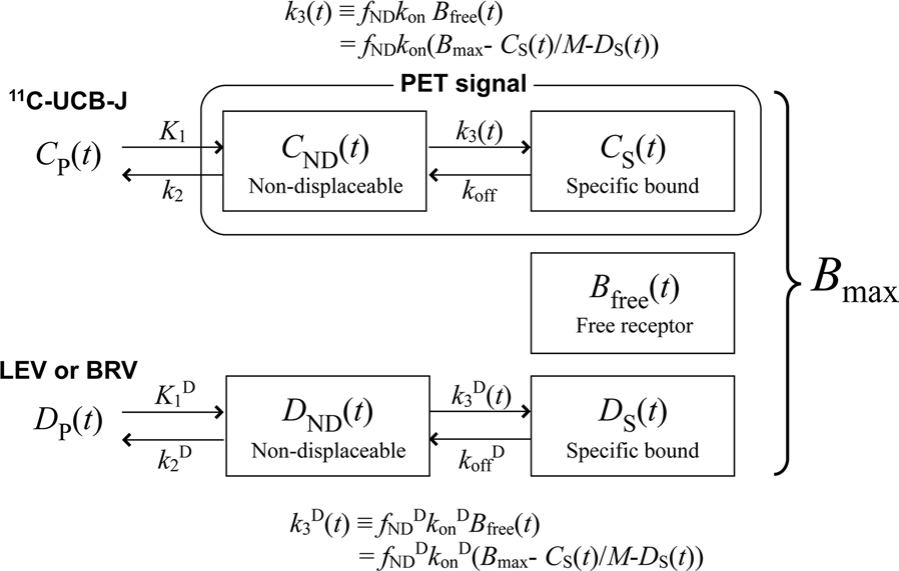
Compartment model for ^11^C-UCB-J and antiepileptic drugs. See Table 1 for definitions. The plasma inputs for tracer and drug (*C*_P_(*t*) and *D*_P_(*t*)) are measured and the sum of *C*_ND_(*t*) and *C*_S_(*t*) is measured as the PET signal. The total SV2A concentration, *B*_max_, is the sum of *C*_S_(*t*) (divided by molar activity), *B*_free_(t), and *D*_S_(*t*)

The compartmental model is illustrated in Fig. 1, and the terminology is defined in Table 1. The blood volume fraction was fixed at 5% (0.05 × whole blood). The proportion of tracer-bound SV2A is assumed to be negligible in comparison with the total SV2A concentration ($B_{{{\text{max}}}}$) so that *C*_s_(*t*)/*M* can be ignored. Using dissociation equilibrium constants of ^11^C-UCB-J and AED and target occupancy *O*(*t*) $\left( { = D_{{\text{S}}} \left( t \right)/B_{{{\text{max}}}} } \right)$, the above model can be expressed as follows:

5 $$\frac{{{\text{d}}C_{{{\text{ND}}}} \left( t \right)}}{{{\text{d}}t}} = K_{1} C_{{\text{P}}} \left( t \right) - k_{2} C_{{{\text{ND}}}} \left( t \right) - k_{{{\text{off}}}} \left\{ {\frac{{f_{{{\text{ND}}}} B_{{{\text{max}}}} }}{{K_{{\text{D}}} }}\left( {1 - O\left( t \right)} \right)C_{{{\text{ND}}}} \left( t \right) - C_{{\text{S}}} \left( t \right)} \right\},$$

6 $$\frac{{{\text{d}}C_{{\text{S}}} \left( t \right)}}{{{\text{d}}t}} = k_{{{\text{off}}}} \left\{ {\frac{{f_{{{\text{ND}}}} B_{{{\text{max}}}} }}{{K_{{\text{D}}} }}\left( {1 - O\left( t \right)} \right)C_{{{\text{ND}}}} \left( t \right) - C_{{\text{S}}} \left( t \right)} \right\},$$

7 $$\frac{{{\text{d}}D_{{{\text{ND}}}} \left( t \right)}}{{{\text{d}}t}} = K_{1}^{{\text{D}}} D_{{\text{P}}} \left( t \right) - k_{2}^{{\text{D}}} D_{{{\text{ND}}}} \left( t \right) - k_{{{\text{off}}}}^{{\text{D}}} B_{{{\text{max}}}} \left\{ {\frac{{f_{{{\text{ND}}}}^{{\text{D}}} }}{{K_{{\text{D}}}^{{\text{D}}} }}\left( {1 - O\left( t \right)} \right)D_{{{\text{ND}}}} \left( t \right) - O\left( t \right)} \right\},$$

8 $$\frac{{{\text{d}}O\left( t \right)}}{{{\text{d}}t}} = k_{{{\text{off}}}}^{{\text{D}}} \left\{ {\frac{{f_{{{\text{ND}}}}^{{\text{D}}} }}{{K_{{\text{D}}}^{{\text{D}}} }}\left( {1 - O\left( t \right)} \right)D_{{{\text{ND}}}} \left( t \right) - O\left( t \right)} \right\}.$$

**Table 1:** Variables and functions and descriptions

Variables and functions	Description
$B_{{{\text{free}}}} \left( t \right)$	Free SV2A concentration in tissue (nmol/L)
$B_{{{\text{max}}}}$	Total SV2A concentration in tissue (nmol/L)
$C_{{{\text{ND}}}} \left( t \right)$	Radioactivity concentration of non-displaceable tracer in tissue (Bq/cm^3^)
$C_{{\text{P}}} \left( t \right)$	Radioactivity concentration of unmetabolized tracer in plasma (Bq/mL)
$C_{{\text{S}}} \left( t \right)$	Radioactivity concentration of specifically bound tracer in tissue (Bq/cm^3^)
$D_{{{\text{ND}}}} \left( t \right)$	Concentration of non-displaceable AED in tissue (nmol/L)
$D_{{\text{P}}} \left( t \right)$	Concentration of unmetabolized AED in plasma (nmol/L)
$D_{{\text{S}}} \left( t \right)$	Concentration of specifically bound AED in tissue (nmol/L)
$f_{{{\text{ND}}}}$	Free fraction of tracer in non-displaceable compartment
$f_{{{\text{ND}}}}^{{\text{D}}}$	Free fraction of AED in non-displaceable compartment
$f_{{\text{P}}}$	Free fraction of tracer in plasma
$f_{{\text{P}}}^{{\text{D}}}$	Free fraction of AED in plasma
$K_{1}$	Rate constant of tracer for transfer from plasma to tissue (mL plasma/min/cm^3^ tissue)
$K_{1}^{{\text{D}}}$	Rate constant of AED for transfer from plasma to tissue (mL plasma/min/cm^3^ tissue)
$k_{2}$	Rate constant of tracer for transfer from tissue to plasma (1/min)
$k_{2}^{{\text{D}}}$	Rate constant of AED for transfer from tissue to plasma (1/min)
$K_{{\text{D}}}$	Dissociation constant of tracer in tissue (nmol/L)
$K_{{\text{D}}}^{{\text{D}}}$	Dissociation constant of AED in tissue (nmol/L)
$k_{{{\text{off}}}}$	Dissociation rate of tracer from SV2A binding sites (1/min)
$k_{{{\text{off}}}}^{{\text{D}}}$	Dissociation rate of AED from SV2A binding sites (1/min)
$k_{{{\text{on}}}}$	Bimolecular association rate constant for tracer (1/nmol/L/min)
$k_{{{\text{on}}}}^{{\text{D}}}$	Bimolecular association rate constant for AED (1/nmol/L/min)
*M*	Molar activity (MBq/µmol)
$O\left( t \right)$	Target occupancy (%)

Introducing the plasma free fractions of ^11^C-UCB-J and AED and the distribution volume of ^11^C-UCB-J, the final model can be expressed as follows:

9 $$\frac{{{\text{d}}C_{{{\text{ND}}}} \left( t \right)}}{{{\text{d}}t}} = K_{1} C_{{\text{P}}} \left( t \right) - \frac{{K_{1} f_{{{\text{ND}}}} }}{{f_{{\text{P}}} }}C_{{{\text{ND}}}} \left( t \right) - k_{{{\text{off}}}} \left\{ {\left( {\frac{{V_{{\text{T}}} f_{{{\text{ND}}}} }}{{f_{{\text{P}}} }} - 1} \right)\left( {1 - O\left( t \right)} \right)C_{{{\text{ND}}}} \left( t \right) - C_{{\text{S}}} \left( t \right)} \right\},$$

10 $$\frac{{{\text{d}}C_{{\text{S}}} \left( t \right)}}{{{\text{d}}t}} = k_{{{\text{off}}}} \left\{ {\left( {\frac{{V_{{\text{T}}} f_{{{\text{ND}}}} }}{{f_{{\text{P}}} }} - 1} \right)\left( {1 - O\left( t \right)} \right)C_{{{\text{ND}}}} \left( t \right) - C_{{\text{S}}} \left( t \right)} \right\},$$

11 $$\frac{{{\text{d}}D_{{{\text{ND}}}} \left( t \right)}}{{{\text{d}}t}} = K_{1}^{{\text{D}}} D_{{\text{P}}} \left( t \right) - \frac{{K_{1}^{{\text{D}}} f_{{{\text{ND}}}}^{{\text{D}}} }}{{f_{{\text{P}}}^{{\text{D}}} }}D_{{{\text{ND}}}} \left( t \right) - k_{{{\text{off}}}}^{{\text{D}}} \frac{{K_{{\text{D}}} }}{{f_{{{\text{ND}}}} }}\left( {\frac{{V_{{\text{T}}} f_{{{\text{ND}}}} }}{{f_{{\text{P}}} }} - 1} \right)\left\{ {\frac{{f_{{{\text{ND}}}}^{{\text{D}}} }}{{K_{{\text{D}}}^{{\text{D}}} }}\left( {1 - O\left( t \right)} \right)D_{{{\text{ND}}}} \left( t \right) - O\left( t \right)} \right\},$$

12 $$\frac{{{\text{d}}O\left( t \right)}}{{{\text{d}}t}} = k_{{{\text{off}}}}^{{\text{D}}} \left\{ {\frac{{f_{{{\text{ND}}}}^{{\text{D}}} }}{{K_{{\text{D}}}^{{\text{D}}} }}\left( {1 - O\left( t \right)} \right)D_{{{\text{ND}}}} \left( t \right) - O\left( t \right)} \right\}.$$

There are 11 unknown parameters in this model: $K_{1} ,{ }f_{{\text{P}}} ,V_{{\text{T}}} ,{ }f_{{{\text{ND}}}} , K_{1}^{{\text{D}}} , f_{{\text{P}}}^{{\text{D}}} , f_{{{\text{ND}}}}^{{\text{D}}} , k_{{{\text{off}}}} , k_{{{\text{off}}}}^{{\text{D}}} , K_{{\text{D}}} ,{\text{and }}K_{{\text{D}}}^{{\text{D}}}$. We proposed to apply the above model to simultaneously fit pairs (displacement and post-dose) of PET scan datasets. Some of these parameters could differ between these two PET scans, and we allowed *K*_1_ and *f*_p_ to vary between the two PET scans. Thus, there were a total of 13 unknown parameters, given the two values for $K_{1}$ and $f_{{\text{P}}}$ for displacement and post-dose PET scans. Literature values and measured values were used to reduce the number of parameters for stable estimation. For ^11^C-UCB-J, *K*_D_ was fixed to 3.4 nM determined in an in vivo self-blocking study in non-human primates [9] and *f*_P_ was measured for each PET scan. For AEDs, $f_{{\text{P}}}^{{\text{D}}}$ was fixed to 0.83 and 0.90 for BRV [18] and LEV [19], respectively. $K_{{\text{D}}}^{{\text{D}}}$ was estimated as the product of these $f_{{\text{P}}}^{{\text{D}}}$ values and *IC*_50_ values from an in vivo SV2A occupancy PET study in humans [4], and then used as the fixed values: 2 µM and 21 µM for BRV and LEV, respectively. $f_{{{\text{ND}}}}^{{\text{D}}}$ was fixed to 0.88 and 1.0 for BRV and LEV, respectively [1]. By using these values, 7 parameters remained to be estimated. In order to further increase stability of the estimation, a 5-parameter (5p) model ($K_{1}$ at displacement and post-dose PET scans,$V_{{\text{T}}} , f_{{{\text{ND}}}} , {\text{and }}K_{1}^{{\text{D}}}$) was also tested where the number of parameters was reduced by fixing the values of $k_{{{\text{off }}}} {\text{and }} k_{{{\text{off}}}}^{{\text{D}}}$ to be averaged across subjects with the 7-parameter (7p) model.

After the 5 parameters were estimated (final estimate), the effect of the selection of fixed parameters on the estimation was tested in two ways. In Test 1, 6-parameter (6p) and 5p models were compared. One parameter out of the fixed parameter set $\left( {f_{{\text{P}}}^{{\text{D}}} , f_{{{\text{ND}}}}^{{\text{D}}} , K_{{\text{D}}} ,K_{{\text{D}}}^{{\text{D}}} , k_{{{\text{off}}}} , {\text{and }}k_{{{\text{off}}}}^{{\text{D}}} } \right)$ and the 5 primary parameters were simultaneously estimated (6p model). The curve fitting error (sum of squares, SS) was compared with that from the 5p model using *F* test. In Test 2, errors in the fixed parameters were assessed. The 5p model was applied by setting 1 fixed parameter to be either a smaller (50%) or bigger (200%) value of the fixed value to see if the estimated values and SS were changed.

For comparison purposes, the one-tissue compartment (1TC) model was applied to the first 60 min of the displacement scan data to estimate *V*_T_ and *K*_1_, which were compared to the matching values from the extended model. Using the LEV and BRV displacement scans, test–retest reproducibility of *V*_T_ and *K*_1_(displacement) was also computed using all models.

A ratio of $K_{1}^{{\text{D}}}$ (BRV $K_{1}^{{\text{D}}}$/LEV $K_{1}^{{\text{D}}}$) was computed from the final estimate to assess the relative delivery of the 2 AEDs. To provide a conservative estimate of the relative speed of brain entry for BRV vs. LEV, a lower bound value on the $K_{1}^{{\text{D}}}$ ratio was determined by finding the lowest BRV $K_{1}^{{\text{D}}}$ and highest LEV $K_{1}^{{\text{D}}}$ that were statistically consistent with the data. The *F* test was used to compare the total of the residual sum of squares of 4 subjects who underwent scans with both AEDs with fixed BRV $K_{1}^{{\text{D}}}$ to that with floating BRV $K_{1}^{{\text{D}}}$ to obtain the lowest statistically feasible BRV $K_{1}^{{\text{D}}}$. The same analysis was performed to find the highest feasible LEV $K_{1}^{{\text{D}}}$. The fixed values of $K_{1}^{{\text{D}}}$ used in this analysis ranged from 30 to 100 µL/cm^3^/min for BRV and 5 to 10 µL/cm^3^/min for LEV, respectively.

All modeling was performed with programs written in MATLAB (MathWorks). Fitting was performed using numerical solutions (ode15s) to these nonlinear differential equations and applied simultaneously to the TACs from the displacement and post-dose scans.

## Results

### Kinetic parameters

Table 2 shows the mean parameters estimated using the 7p and 5p models and their standard errors for each study. The primary PET tracer parameters *K*_1_ and *V*_T_ from the extended models were in good agreement with those derived from the conventional one-tissue compartment model using the first 60 min of data from each displacement scan (Additional file 1: Table S1). *V*_T_ values from the 7p and 5p models were lower than the 1TC values by 3 ± 4% and 4 ± 4%, respectively, while *K*_1_ values were higher by 6 ± 5% and 7 ± 3% excluding one case (40% higher in the cerebellum for *K*_1_ (LEV displacement) with the 7p model). The test–retest reproducibility of *K*_1_ and *V*_T_ was similar among all models (Table 3). In addition, allowing *K*_1_ to change at the time of displacement (mid-way through the scan) did not alter values of *K*_1_ or $K_{1}^{{\text{D}}}$ (data not shown).

**Table 2:** Kinetic parameters estimated using the 7-parameter and 5-parameter models (LEV: *n* = 4, BRV: *n* = 5)

Model	Region	AED			^11^C-UCB-J				
		AED	*K*_1_^D^(µL/cm^3^/min)	*k*_off_^D^(1/min)	*V*_T_ (mL/cm^3^)	*f*_ND_	*K*_1_ (mL/cm^3^/min)		*k*_off_(1/min)
							Displacement	Post-dose	
7-parameter	Putamen	LEV	6.2 (45%)	4.2 (47%)	22.2 (5%)	0.075 (13%)	0.46 (5%)	0.49 (8%)	5.5 (31%)
	Frontal cortex	LEV	5.3 (42%)	4.9 (71%)	18.9 (5%)	0.087 (13%)	0.43 (5%)	0.44 (7%)	4.6 (39%)
	Cerebellum	LEV	4.8 (81%)	8.9 (679%)	13.8 (5%)	0.093 (14%)	0.41 (5%)	0.38 (6%)	3.4 (32%)
	Putamen	BRV	75.3 (86%)	9.2 (271%)	21.5 (6%)	0.087 (31%)	0.46 (6%)	0.45 (12%)	8.5 (40%)
	Frontal cortex	BRV	321.6 (99%)	9.0 (133%)	18.6 (6%)	0.098 (27%)	0.44 (7%)	0.41 (20%)	5.4 (37%)
	Cerebellum	BRV	58.9 (91%)	10.1 (923%)	13.9 (6%)	0.096 (23%)	0.37 (6%)	0.35 (15%)	8.8 (34%)
5-parameter	Putamen	LEV	5.2 (42%)		21.6 (4%)	0.076 (11%)	0.47 (3%)	0.49 (5%)	
	Frontal cortex	LEV	5.1 (41%)		18.7 (4%)	0.088 (11%)	0.43 (3%)	0.43 (5%)	
	Cerebellum	LEV	4.0 (59%)		13.7 (4%)	0.099 (11%)	0.37 (4%)	0.36 (6%)	
	Putamen	BRV	88.3 (46%)		21.5 (5%)	0.089 (27%)	0.46 (4%)	0.46 (10%)	
	Frontal cortex	BRV	72.9 (49%)		18.3 (5%)	0.105 (26%)	0.44 (4%)	0.42 (10%)	
	Cerebellum	BRV	43.9 (57%)		13.5 (5%)	0.094 (21%)	0.37 (4%)	0.35 (11%)	

Data are median parameter estimates (median percent standard error) for AEDs and mean parameter estimates (mean percent standard error) for the ^11^C-UCB-J

**Table 3:** Test–retest reproducibility of binding parameters

Region	*V*_T_ (mL/cm^3^) (*n* = 4)			*K*_1_ (mL/cm^3^/min) (*n* = 4)		
	7-parameter model	5-parameter model	1TC	7-parameter	5-parameter	1TC
Putamen	− 4 ± 6%	− 1 ± 6%	2 ± 9%	− 2 ± 9%	− 3 ± 8%	− 4 ± 8%
Frontal cortex	− 2 ± 7%	− 2 ± 6%	0 ± 7%	− 2 ± 9%	− 2 ± 10%	− 2 ± 11%
Cerebellum	3 ± 12%	0 ± 9%	2 ± 7%	*− 5 ± 10%	− 2 ± 8%	− 3 ± 6%

Data are mean and SD of test–retest reproducibility computed using $\left({\text{test}}-{\text{retest}}\right)/\left({\text{test}}+{\text{retest}}\right)\times 2$

*n = 3

With the 7p model (Table 2), the range of $K_{1}^{{\text{D}}}$ values was small for LEV (4.8–6.2 µL/cm^3^/min) across regions with 45% median relative standard error (rSE), but large (59–322 µL/cm^3^/min) for BRV and unstably estimated with large rSE (median: 91%). The dissociation rate $k_{{{\text{off}}}}^{{\text{D}}}$ also had larger standard errors for both AEDs, especially for BRV. $k_{{{\text{off}}}}$ for ^11^C-UCB-J was estimated more reliably than $k_{{{\text{off}}}}^{{\text{D}}}$; however, average %SE values were 30–40%. The mean values (1/min), excluding estimates with rSE > 100%, were 4.9 (*n* = 25), 4.9 (*n* = 7), and 5.9 (*n* = 2) for ^11^C-UCB-J $k_{{{\text{off}}}}$, LEV $k_{{{\text{off}}}}^{{\text{D}}}$, and BRV $k_{{{\text{off}}}}^{{\text{D}}}$, respectively. These *k*_off_ values correspond to dissociation half-times of less than 10 s and are thus not numerically identifiable.

For LEV, the estimated parameters by the 7p and 5p models were similar, excluding one case (the same one described in comparison with the one-tissue compartment model). For both AEDs, the SS remained unchanged between models; thus, the *F* test to compare the two models showed that the 7p model was not significantly better than the 5p model. LEV $K_{1}^{{\text{D}}}$ was decreased slightly with the 5p model by 7% (median), and BRV $K_{1}^{{\text{D}}}$ was decreased by 9%. The rSE of $K_{1}^{{\text{D}}}$ decreased in all regions by 38% (BRV $K_{1}^{{\text{D}}}$) and 2% (LEV $K_{1}^{{\text{D}}}$). Thus, the 5p model provided more reliable estimates of BRV and LEV $K_{1}^{{\text{D}}}$, although %SE values were still high (~ 50%).

**Fig. 2:**
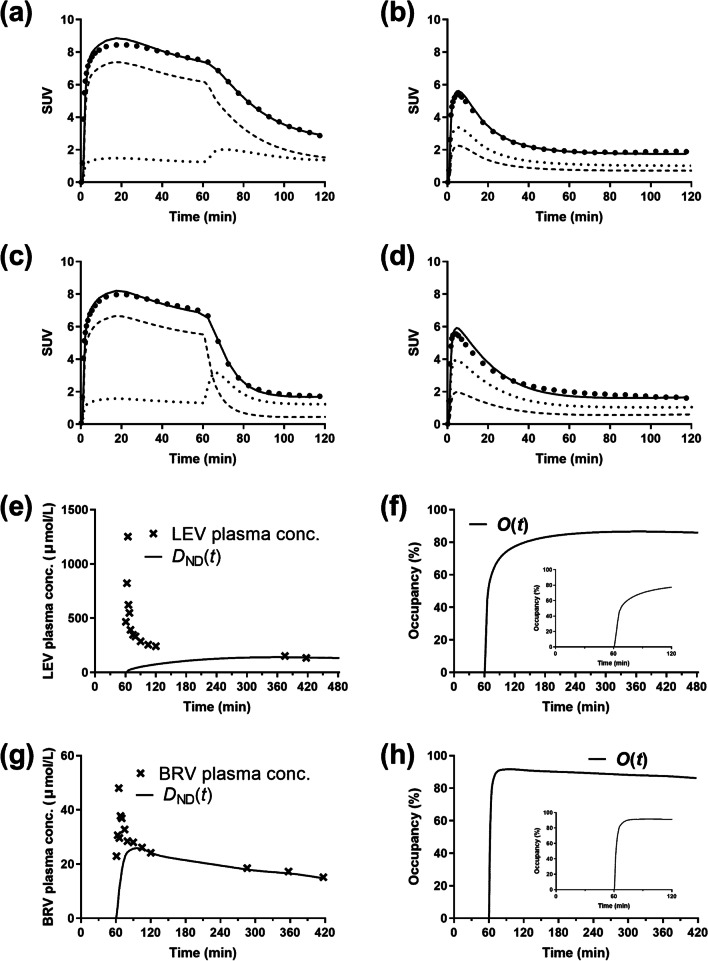
**a**–**d** ^11^C-UCB-J activity curves in the frontal cortex (closed circles) with model fits (solid curves). **a** and **b** Displacement (LEV, 1500 mg at 60 min) and post-dose scans, **c** and **d** displacement (BRV, 200 mg at 60 min) and post-dose scans. *C*_ND_(*t*) and *C*_S_(*t*) are displayed in the dotted and dashed curves, respectively. **e**–**h** Concentrations of AED in the plasma and non-displaceable AED in the frontal cortex (*D*_ND_(*t*)) and occupancy curves (*O*(*t*)) by LEV (**e**, **f**) and BRV (**g**, **h**). Insets in **f** and **h** show the occupancy curves for the first 2 h

Figure 2a–d shows examples of curve fitting in the frontal cortex using the 5-parameter model. In both displacement and post-dose scans, there was a tendency for the model fit to exceed the early data points for all subjects. Examples of curve fittings in the putamen and cerebellum are shown in Additional file 1: Figs. S1 and S2. Figure 2e–h shows the AED concentration curves in plasma and brain tissue and occupancy curves in the frontal cortex. Examples of the AED concentration curves and occupancies in the putamen and cerebellum are shown in Additional file 1: Figs. S3 and S4. The concentrations of BRV in plasma and model-predicted non-displaceable BRV in the brain were similar at 20–40 min after BRV administration (97 ± 10% at 30 min), while the concentration of non-displaceable LEV in the brain was still lower than the plasma LEV concentration at 1 h after LEV administration (32 ± 6%). The model-predicted peak occupancy and the time to reach peak occupancy after AED administration are shown in Table 4. The occupancy by LEV gradually increased until 4.6 ± 0.8 h after administration, while the occupancy by BRV reached its peak at 27 ± 14 min after administration and then slowly decreased.

**Table 4:** Maximum target occupancies by AEDs and time to reach maximum and half occupancy after AED administration

Drug	Dose (mg)	Maximum occupancy (%)	Time to reach maximum occupancy (min)	Time to reach half occupancy (min)
LEV	1500	84 ± 2	277 ± 48	6.8 ± 0.8
BRV	50	63 ± 1	38 ± 10	4.1 ± 0.3
BRV	100	81 ± 3	21 ± 14	3.2 ± 0.4
BRV	200	92 ± 2	29 ± 15	1.9 ± 0.5

The sensitivity of the pre-selected parameters in the 5p fit was evaluated with 2 tests. In Test 1 (adding one additional floating parameter), for both AEDs, all curve fits using the 6p model were not significantly better than those using the 5p model. In the 6p models, the estimates of $K_{1}^{{\text{D}}}$ became unstable (rSE > 100%), especially when any of BRV $f_{{{\text{ND}}}}^{{\text{D}}}$, LEV $f_{{{\text{ND}}}}^{{\text{D}}}$, or BRV $k_{{{\text{off}}}}^{{\text{D}}}$ was set as a floating parameter. In Test 2 (altering fixed parameters by − 50% or + 100%), the estimates of *V*_T_ and *K*_1_ were not affected by changing any of the fixed values for either AED. Changing the fixed values for either $K_{{\text{D}}}^{{\text{D}}}$ or $f_{{\text{P}}}^{{\text{D}}}$ greatly affected the estimates of *f*_ND_ and $K_{1}^{{\text{D}}}$. Changing $f_{{{\text{ND}}}}^{{\text{D}}}$ affected only the estimates of $K_{1}^{{\text{D}}}$. Table 5 shows the mean and SD of percent change of the estimated parameters and SS across regions when one of the fixed parameters was set to either 50% or 200% of the fixed values in the 5p model. For LEV, $K_{1}^{{\text{D}}}$ was underestimated with 50% $K_{{\text{D}}}^{{\text{D}}}$ and overestimated with 200% $K_{{\text{D}}}^{{\text{D}}}$ in all subjects. However, for BRV, $K_{1}^{{\text{D}}}$ estimates varied across subjects when changing the fixed values of $K_{{\text{D}}}^{{\text{D}}}$ (e.g., − 22 to 124% with 50% $K_{{\text{D}}}^{{\text{D}}}$ and − 59 to 10% with 200% $K_{{\text{D}}}^{{\text{D}}}$ in the frontal cortex). The curve fitting was poor for the BRV displacement scans with 200% $K_{{\text{D}}}^{{\text{D}}}$ and 50% $f_{{\text{P}}}^{{\text{D}}}$ in three subjects, i.e., the model fits fell below the data from 30 to 70 min post-injection. For both AEDs, errors in the assumed values of $k_{{{\text{off}}}}$, $k_{{{\text{off}}}}^{{\text{D}}}$, and $K_{{\text{D}}}$ had a minimal effect on $K_{1}^{{\text{D}}}$; errors in *f*_P_ (assuming ± 25%) also had a minimal effect (− 5 to 2%).

**Table 5:** Percent change of the estimated parameters and curve fitting error by 50% and 200% of the fixed values

Drug	Parameter	$k_{{{\text{off}}}}$	$k_{{{\text{off}}}}$	$k_{{{\text{off}}}}^{{\text{D}}}$	$k_{{{\text{off}}}}^{{\text{D}}}$	$K_{{\text{D}}}$	$K_{{\text{D}}}$	$K_{{\text{D}}}^{{\text{D}}}$	$K_{{\text{D}}}^{{\text{D}}}$	$f_{{\text{P}}}^{{\text{D}}}$	$f_{{{\text{ND}}}}^{{\text{D}}}$
		50%	200%	50%	200%	50%	200%	50%	200%	50%	50%
LEV	$V_{{\text{T}}}$	0 ± 1	0 ± 1	0 ± 1	0 ± 1	0 ± 1	0 ± 1	1 ± 1	− 2 ± 1	0 ± 1	− 1 ± 1
LEV	$f_{{{\text{ND}}}}$	− 1 ± 2	0 ± 1	− 2 ± 3	0 ± 2	0 ± 1	0 ± 1	− 21 ± 5	134 ± 78	145 ± 96	0 ± 1
LEV	$K_{1} \left( {{\text{disp}}.} \right)$	1 ± 1	0 ± 0	0 ± 1	0 ± 1	0 ± 0	0 ± 0	− 1 ± 1	1 ± 1	0 ± 1	0 ± 0
LEV	$K_{1} \left( {{\text{post}}.} \right)$	1 ± 0	0 ± 0	0 ± 0	0 ± 0	0 ± 0	0 ± 0	0 ± 0	1 ± 1	1 ± 1	0 ± 0
LEV	$K_{1}^{{\text{D}}}$	1 ± 6	− 1 ± 6	2 ± 9	− 1 ± 6	0 ± 4	0 ± 5	− 37 ± 5	44 ± 7	− 24 ± 6	96 ± 9
LEV	SS	0 ± 0	0 ± 0	0 ± 1	0 ± 0	0 ± 0	0 ± 0	1 ± 0	0 ± 1	0 ± 1	0 ± 0
BRV	$V_{{\text{T}}}$	− 1 ± 2	− 1 ± 3	0 ± 2	0 ± 1	0 ± 2	0 ± 2	2 ± 3	− 7 ± 7	− 7 ± 7	0 ± 2
BRV	$f_{{{\text{ND}}}}$	− 3 ± 7	− 2 ± 10	1 ± 7	2 ± 8	1 ± 7	1 ± 7	− 34 ± 18	253 ± 208	266 ± 215	− 1 ± 9
BRV	$K_{1} \left( {{\text{disp}}.} \right)$	1 ± 1	0 ± 1	0 ± 1	0 ± 1	0 ± 1	0 ± 1	− 1 ± 1	3 ± 3	3 ± 3	0 ± 1
BRV	$K_{1} \left( {{\text{post}}.} \right)$	0 ± 1	0 ± 0	0 ± 1	0 ± 1	0 ± 1	0 ± 1	1 ± 2	− 2 ± 3	− 2 ± 3	0 ± 0
BRV	$K_{1}^{{\text{D}}}$	− 2 ± 12	− 5 ± 11	2 ± 9	− 2 ± 10	− 3 ± 15	− 1 ± 11	58 ± 96	− 18 ± 33	− 57 ± 15	87 ± 27
BRV	SS	0 ± 0	0 ± 0	0 ± 0	0 ± 0	0 ± 0	0 ± 0	0 ± 1	6 ± 6	6 ± 6	0 ± 0

Data are mean and SD of percent change compared with the estimated parameter using the 5p model. A positive value means that the estimated parameter using the 5p model with either 50% or 200% of the fixed value is higher than that using the 5p model

### $K_{1}^{{\text{D}}}$ratio

The BRV/LEV $K_{1}^{{\text{D}}}$ ratios from the 5p fits were 17.1 (putamen), 13.7 (frontal), and 10.8 (cerebellum). However, due to the large uncertainty in the LEV and BRV $K_{1}^{{\text{D}}}$ values, a lower bound value on the ratio of BRV/LEV $K_{1}^{{\text{D}}}$ was determined to provide a conservative estimate of the relative entry of BRV and LEV. This bound value was calculated by finding the lowest BRV $K_{1}^{{\text{D}}}$ and highest LEV $K_{1}^{{\text{D}}}$ that were statistically consistent with the TACs. Curve fitting was significantly different (*P* < 0.05) when $K_{1}^{{\text{D}}}$ (µL/cm^3^/min) of LEV was higher than 5.53 (putamen), 5.91 (frontal), and 4.78 (cerebellum) or $K_{1}^{{\text{D}}}$ (µL/cm^3^/min) of BRV was lower than 42.3 (putamen), 49.2 (frontal), and 23.0 (cerebellum). Therefore, the ratio of $K_{1}^{{\text{D}}}$ (BRV/LEV) was higher than 7.6 (putamen), 8.3 (frontal), and 4.8 (cerebellum), or at least ~ 7 on average.

## Discussion

In this displacement study, the extended compartment model accounting for dynamic changes in receptor occupancy was applied to estimate the kinetic parameters of ^11^C-UCB-J and AEDs simultaneously. The kinetic parameters of AEDs, especially the drug uptake constant $K_{1}^{{\text{D}}}$, were the parameters of most interest. This extended model used 13 parameters to describe the relationship between the ^11^C-UCB-J PET data and the time-varying plasma concentration of AEDs. The number of parameters was initially reduced to 7 to stabilize the estimates using literature values and measured values. However, the 7p model resulted in unstable estimates of AED-related parameters, especially for BRV, likely due to the more rapid kinetics. Therefore, based on the stability of parameters and the results of the *F* test, fixing the values of $k_{{{\text{off }}}} {\text{and }}k_{{{\text{off}}}}^{{\text{D}}}$ with the average across subjects, the 5p model was selected as the best model. Results with this model showed that BRV occupied SV2A sites faster than LEV. BRV $K_{1}^{{\text{D}}}$ was approximately sevenfold higher than LEV $K_{1}^{{\text{D}}}$.

Human ^11^C-UCB-J brain PET data from scans without displacement have been successfully fitted with the one-tissue compartment model [5, 20, 21]. The addition of non-equilibrium conditions in this study allowed the use of an extended model, which is based on the two-tissue compartment model. The extended model described well both the displacement and post-dose ^11^C-UCB-J TACs. However, regardless of the type of AED, there was a lack of fit at ~ 20 min after administration in all regions and subjects. It is possible that the extended model is insufficient to describe the kinetics of ^11^C-UCB-J competing with AEDs. For example, some fixed values were derived from studies of non-human primates; these values may differ in humans and such differences may have affected the estimates. When the *K*_D_ of ^11^C-UCB-J was fixed to the literature value, the implementation of the extended model is effectively a two-tissue three-parameter compartment model for ^11^C-UCB-J. Even with this simplified model, the %SE of *f*_ND_ was relatively large, especially in the case of displacement with BRV, suggesting the limited ability of this modeling approach to extract more kinetic information.

Sampling of AED levels in blood is an important issue that affects modeling success. The primary goal of pharmacokinetic samples is for comparison to PET-derived occupancy values. Here, we attempted to use these data as the AED input function, $D_{{\text{P}}} \left( t \right)$. However, there was only limited sampling during the first 5 min after the start of the AED infusion, with only 1–3 samples. Thus, the quality of curve fitting may have been deteriorated due to insufficient sampling frequency. Increasing the frequency of blood measurements or applying established pharmacological models to obtain accurate AED input functions would improve the quality of curve fitting and the estimation of $K_{1}^{{\text{D}}}$. Also, this lack of fit remained even when all parameters were allowed to float, suggesting that the quality of curve fitting was not degraded due to the fixed parameters. Although some lack of fit was observed, the estimated parameters, *K*_1_ and *V*_T_, agreed well with those from the 1TC model.

We evaluated the effects of the fixed parameters on the kinetic estimates by changing them individually (Test 2). In the extended model, altering the fixed AED-related parameters greatly affected the estimates of $K_{1}^{{\text{D}}}$. BRV $K_{1}^{{\text{D}}}$ estimation was more unstable than LEV $K_{1}^{{\text{D}}}$ estimation, likely due to its higher speed of brain entry. For the LEV-related parameters, if $K_{{\text{D}}}^{{\text{D}}}$ is reduced (increasing affinity of AED), then less of the AED is needed in the brain to displace the tracer, thus leading to a reduced $K_{1}^{{\text{D}}}$. However, other relationships ($K_{{\text{D}}}^{{\text{D}}}$ and $f_{{\text{P}}}^{{\text{D}}}$ vs. *f*_ND,_ $f_{{\text{P}}}^{{\text{D}}}$ and $f_{{{\text{ND}}}}^{{\text{D}}}$ vs. $K_{1}^{{\text{D}}}$) cannot be explained in a straightforward manner. This is especially true given the slight lack of fit to the data at ~ 20 min (Fig. 2), so this portion of the data may have an overly large effect on the changes in the floating parameters to account for changes in the fixed parameter.

There have been several studies using kinetic models to describe the kinetics of tracer and unlabeled tracer/drug blocking the receptor simultaneously. The model equations we proposed are essentially the same as those used by Delforge et al. [16] and Endres et al. [17]. The difference between our study and the ^11^C-flumazenil study of Delforge et al. is that the benzodiazepine receptors were blocked by the unlabeled tracer. In that case, since all kinetic parameters and affinities were the same between labeled and unlabeled tracers, the number of parameters was 6, much less than in our model (13 parameters including the two measured values of *f*_P_). The input function of the unlabeled tracer was assumed to be similar in shape and to be proportional to the injected tracer mass, resulting in an input function with the same sample timing as that for the tracer. In the ^11^C-raclopride study by Endres et al., amphetamine was administered to stimulate dopamine release. Microdialysis samples were taken to derive free dopamine in the brain. The difference from our proposed model is that they directly measured free dopamine in the brain instead of plasma concentration, and thus, the drug entry rate, $K_{1}^{{\text{D}}}$, was not included in the model. They also assumed that bound dopamine was always in equilibrium with synaptic free dopamine. These differences resulted in models with 5 or 6 parameters.

Another approach described makes use of a reference tissue model. A study by Johansson et al. [22] used a reference tissue model (lp-ntPET2 [23]) to estimate the time-dependent changes in mu-opioid receptor availability (*BP*_ND_) by intranasal naloxone. The bound drug concentration was described by a gamma-variate function in the model and the drug entry rate was not included in the model. In a study with ^11^C-DASB and duloxetine by Abanades et al. [24], it was assumed that there was instantaneous equilibration between the plasma and the brain tissue and a finite rate of exchange between tissue free drug concentration and receptors. A relationship between drug plasma concentration and occupancy was described with two parameters ($k_{{{\text{off}}}}^{{\text{D}}}$ and $k_{{{\text{on}}}}^{{\text{D}}}$). This model is useful when there is a pharmacological hysteresis due to a dynamic lag between free in brain tissue and target bound concentrations [25]. In contrast, our extended model is useful when there is hysteresis due to slow entry of drug into brain ($K_{1}^{{\text{D}}} ).$ Comparing AED plasma concentrations and anticonvulsant activity values in mice, LEV peak activity was delayed by ~ 1 h relative to peak plasma levels, whereas BRV did not show hysteresis. When anticonvulsant activity was compared with brain drug level rather than plasma levels, the hysteresis in the PK/PD plot disappeared [1]. The difference in the rate of onset of action is reflected in the difference in $K_{1}^{{\text{D}}}$ between BRV and LEV.

The brain entry half-times of these AEDs were previously estimated in a simpler analysis by modeling the tracer displacement curve with a single exponential model [4]. With higher doses of BRV, the brain entry half-time was shorter (10 min for 100 mg BRV, *n* = 2; 2 min for 200 mg, *n* = 2). In addition, the BRV brain entry half-time was faster than that of LEV (20 min for 1500 mg, *n* = 6). In the present analysis using the extended model, the time to reach half occupancy after AED administration (Table 4) was shorter at the higher doses of BRV, and the time to reach half occupancy by LEV was approximately twice that of BRV. There was a greater difference in the time to reach maximum occupancy between the two AEDs (9 times); however, the estimates of the time to reach maximum occupancy were less precise than those of the time to reach half occupancy.

## Conclusions

In summary, the extended compartment model allowed us to directly estimate the rate of entry of the AEDs into the brain tissue and to estimate the change in SV2A occupancy over time. This analysis reconfirmed that BRV enters brain tissue faster than LEV, by at least a factor of 7. This approach extends the capabilities of PET imaging to not only examine drug target occupancy, but also drug uptake. However, this approach may only be applicable to orally administered drugs with very rapid absorption or those administered intravenously, like BRV and LEV, intranasally, like naloxone, or the investigational inhaled powder Staccato alprazolam.

## Supplementary Information

**Additional file 1: Fig. S1.** ^11^C-UCB-J activity curves in putamen (closed circles) with model fits (solid curves). **a** and **b** Displacement (LEV, 1500 mg at 60 min) and post-dose scans, **c** and **d** displacement (BRV, 200 mg at 60 min) and post-dose scans. *C*_ND_(*t*) and *C*_S_(*t*) was displayed in the dotted curves and break curves, respectively. **Fig. S2.** ^11^C-UCB-J activity curves in cerebellum (closed circles) with model fits (solid curves). **a** and **b** displacement (LEV, 1500 mg at 60 min) and post-dose scans, **c** and **d** displacement (BRV, 200 mg at 60 min) and post-dose scans. *C*_ND_(*t*) and *C*_S_(*t*) was displayed in the dotted curves and break curves, respectively. **Fig. S3.** Concentrations of AED in the plasma and non-displaceable AED in the putamen (*D*_ND_(*t*)) and occupancy curves by LEV (**a**, **b**) and BRV (**c**, **d**). Insets in (**b**) and (**d**) show the occupancy curves for the first 2 h. **Fig. S4.** Concentrations of AED in the plasma and non-displaceable AED in the cerebellum (*D*_ND_(*t*)) and occupancy curves by LEV (**a**, **b**) and BRV (**c**, **d**). Insets in (**b**) and (**d**) show the occupancy curves for the first 2 h. **Table S1.** Kinetic parameters estimated using the one-tissue compartment model (LEV: *n* = 4, BRV: *n* = 5).

## Data Availability

The datasets used and analyzed during the current study are available from the corresponding author upon reasonable request.

## Abbreviations

AAL: Automated anatomical labeling
AED: Antiepileptic drug
BRV: Brivaracetam
HRRT: High-resolution research tomograph
LEV: Levetiracetam
MNI: Montreal Neurological Institute
MR: Magnetic resonance
PET: Positron emission tomography
ROI: Region of interest
SS: Sum of squares
SV2A: Synaptic vesicle glycoprotein 2A
TAC: Time-activity curve
1TC: One-tissue compartment
