# Supplementary material for: Drug characteristics derived from kinetic modeling: combined 11C-UCB-J human PET imaging with levetiracetam and brivaracetam occupancy of SV2A
Source: EJNMMI Res. 2022 Nov 8;12:71. doi: 10.1186/s13550-022-00944-5 (PMC9643320; doi:10.1186/s13550-022-00944-5)
Supplement: Supplementary file 1 — Additional file 1: Fig. S1. 11C-UCB-J activity curves in putamen (closed circles) with model fits (solid curves). a and b Displacement (LEV, 1500 mg at 60 min) and post-dose scans, c and d displacement (BRV, 200 mg at 60 min) and post-dose scans. CND(t) and CS(t) was displayed in the dotted curves and break curves, respectively. Fig. S2. 11C-UCB-J activity curves in cerebellum (closed circles) with model fits (solid curves). a and b displacement (LEV, 1500 mg at 60 min) and post-dose scans, c and d displacement (BRV, 200 mg at 60 min) and post-dose scans. CND(t) and CS(t) was displayed in the dotted curves and break curves, respectively. Fig. S3. Concentrations of AED in the plasma and non-displaceable AED in the putamen (DND(t)) and occupancy curves by LEV (a, b) and BRV (c, d). Insets in (b) and (d) show the occupancy curves for the first 2 h. Fig. S4. Concentrations of AED in the plasma and non-displaceable AED in the cerebellum (DND(t)) and occupancy curves by LEV (a, b) and BRV (c, d). Insets in (b) and (d) show the occupancy curves for the first 2 h. Table S1. Kinetic parameters estimated using the one-tissue compartment model (LEV: n = 4, BRV: n = 5). [file 13550_2022_944_MOESM1_ESM.docx]

# Additional file 1

#



**Supplementary Figure 1** ^11^C-UCB-J activity curves in putamen (closed circles) with model fits (solid curves). (a) and (b) displacement (LEV, 1500 mg at 60 min) and post-dose scans, (c) and (d) displacement (BRV, 200 mg at 60 min) and post-dose scans. *C*_ND_(*t*) and *C*_S_(*t*) was displayed in the dotted curves and break curves, respectively.



**Supplementary Figure 2** ^11^C-UCB-J activity curves in cerebellum (closed circles) with model fits (solid curves). (a) and (b) displacement (LEV, 1500 mg at 60 min) and post-dose scans, (c) and (d) displacement (BRV, 200 mg at 60 min) and post-dose scans. *C*_ND_(*t*) and *C*_S_(*t*) was displayed in the dotted curves and break curves, respectively.

#



**Supplementary Figure 3** Concentrations of AED in the plasma and non-displaceable AED in the putamen (*D*_ND_(*t*)) and occupancy curves by LEV ((a) and (b)) and BRV ((c) and (d)). Insets in (b) and (d) show the occupancy curves for the first 2 h.

**

 Supplementary Figure 4** Concentrations of AED in the plasma and non-displaceable AED in the cerebellum (*D*_ND_(*t*)) and occupancy curves by LEV ((a) and (b)) and BRV ((c) and (d)). Insets in (b) and (d) show the occupancy curves for the first 2 h.

# Supplementary Table 1: Kinetic parameters estimated using the one-tissue compartment model (LEV: *n* = 4, BRV: *n* = 5)

| Region | AED | *V*_T_ (mL/cm^3^) | | *K*_1_ (mL/cm^3^/min) | |
| --- | --- | --- | --- | --- | --- |
|  |  | displacement | post-dose | displacement | post-dose |
| Putamen | LEV | 22.6 ± 1.1 | 6.9 ± 0.6 | 0.44 ± 0.04 | 0.45 ± 0.04 |
| Frontal cortex | LEV | 19.4 ± 0.6 | 6.0 ± 0.6 | 0.41 ± 0.05 | 0.40 ± 0.03 |
| Cerebellum | LEV | 13.8 ± 1.1 | 4.9 ± 0.2 | 0.35 ± 0.07 | 0.33 ± 0.06 |
| Putamen | BRV | 23.3 ± 2.1 | 8.7 ± 2.8 | 0.43 ± 0.06 | 0.44 ± 0.04 |
| Frontal cortex | BRV | 19.5 ± 1.4 | 7.4 ± 2.4 | 0.41 ± 0.07 | 0.39 ± 0.03 |
| Cerebellum | BRV | 14.0 ± 0.7 | 6.1 ± 1.3 | 0.34 ± 0.07 | 0.32 ± 0.07 |

Data are mean and SD of parameter estimates
